# Comprehensive geriatric assessment in perioperative care: a protocol for a systematic review and qualitative synthesis

**DOI:** 10.1136/bmjopen-2021-049875

**Published:** 2021-12-28

**Authors:** Rachael Lucia Miller, Jonathan David Barnes, Ronelle Mouton, Philip Braude, Robert Hinchliffe

**Affiliations:** 1 Translational Health Sciences, University of Bristol, Bristol, UK; 2 Vascular Surgery, Musgrove Park Hospital, Taunton, UK; 3 Anaesthesia, North Bristol NHS Trust, Bristol, UK; 4 CLARITY (Collaborative Ageing Research) group, Department of Medicine for Older People, North Bristol NHS Trust, Bristol, UK; 5 Vascular Surgery, North Bristol NHS Trust, Bristol, UK

**Keywords:** geriatric medicine, adult surgery, protocols & guidelines

## Abstract

**Introduction:**

Comprehensive geriatric assessment (CGA) is an intervention that has been deployed in the perioperative setting with the aim to improve outcomes for older patients admitted to hospital. Older patients undergoing surgery are more likely to have postoperative complications, a longer hospital stay and be discharged to a care facility. Despite the increasing application of this intervention within surgical services, the evidence for CGA remains limited in this group. The aim of this systematic review is to describe CGA as in intervention applied to surgical populations in randomised controlled trials (RCTs) as well as the outcomes assessed.

**Methods and analysis:**

A systematic search of RCTs of CGA in surgery will be run in Embase, Medline, CINAHL (Cumulative Index to Nursing and Allied Health Literature) and Cochrane library. Further articles will be identified from reference lists in relevant studies found in the search. A narrative synthesis will be undertaken outlining specialties included, detailed descriptions of the intervention and outcomes.

**Ethics and dissemination:**

No ethical approval is required. The results of this review will be published and used as the basis of work to optimise this intervention for future trials in surgical populations.

**PROSPERO registration number:**

This review is registered with PROSPERO CRD42020221797.

**Strengths and limitations of this study**

- This will be the first methodological, systematic review to conduct a qualitative analysis and summarise the reporting of comprehensive geriatric assessment (CGA) as an intervention in perioperative care.
- Only randomised controlled trials evaluating CGA in the perioperative period as an intervention will be included.
- This review will not report a meta-analysis of quantitative results.
- This review will describe how trials report CGA standard of care, a novel aspect.

## Introduction

### Rationale

The average age of surgical patients is increasing bringing novel challenges to healthcare professionals within the perioperative pathway.^1 2^ Compared with younger patients, older people have a higher postoperative mortality and are more likely to experience significant postoperative complications, longer length of hospital stay and greater likelihood of discharge to a care facility.^3^ For example, according to the latest report from the National Emergency Laparotomy Audit, the 30-day mortality in patients over 65 years old and living with frailty was considerably above average at 18% compared with the overall 9.3% for this surgery.^1^

Comprehensive geriatric assessment (CGA) has been employed to improve outcomes for older patients admitted to hospital. Originally described in the 1930s, descriptions and practice of CGA have varied widely in the literature.^4^ CGA is frequently defined as a ‘multidimensional diagnostic and therapeutic process that is focused on determining a frail older person’s medical, functional, mental, and social capabilities and limitations with the goal of ensuring that problems are identified, quantified, and managed appropriately’.^5^ It has been widely adopted in the care of the hospitalised older person, with an associated reduction in 1-year mortality and institutionalisation posthospital discharge.^5^ Evidence of benefit within surgical populations is more limited and have focused mainly on patients who need surgery for hip fracture.^6^ The most recent Cochrane review on perioperative CGA lacked generalisability to all surgical disciplines due to the limited populations the randomised trials included: seven trials in hip fracture, and one in elective surgical oncology.^6^ Since the search was conducted in January 2017 further trials have been completed in other surgical specialties. While the Cochrane review focused on the health outcomes of CGA in a perioperative setting, this protocol describes a systematic review that will develop the existing knowledge by focussing on qualitative analysis of the literature, paying particular attention to the timing, components and team members involved in the intervention.

There is currently significant variation in how CGA is defined and reported in clinical research with no robustly developed consensus definitions.^7–9^ Definitions of perioperative CGA vary from which multidisciplinary team members should be included, which domains should be assessed and optimised, when is the right point of delivery (preoperatively or postoperatively) and even which patients should be selected.^8^ This provides a lack of standardisation in delivery of CGA and which aspects could be strengthened, or removed, to increase the efficacy of this complex intervention to achieve positive outcomes.^10^ One recent review has attempted to outline the core components of CGA in medical patients.^8^ However, no study has fully laid out the features of trial design or analysed the variation of delivery of this intervention for surgical patients.^1^

This protocol is designed to systematically review and summarise the reporting of CGA as an intervention in perioperative randomised controlled trials (RCTs). It will be reported according to Preferred Reporting Items for Systematic Review and Meta-Analysis Protocols statement (online supplemental information 1).^11^

10.1136/bmjopen-2021-049875.supp1 Supplementary data

### Aim

The aim of this systematic review is to describe CGA as in intervention applied to surgical populations in RCTs.

#### Specific objectives

1. Examine the described components of CGA as an intervention in identified trials, including how, when and by whom these are delivered.
2. Identify surgical populations where randomised controlled studies have been performed comparing CGA to any other care, in either an elective or emergency surgical population.
3. Describe how trials report ‘standard care’.
4. Determine what outcomes have been used to assess effectiveness of CGA and whether these reflect a biological plausibility of how CGA affects outcomes

### Methods

Data item numbers collected include:

1. Participants: sex, age, number randomised, target sample size, reasons for non-recruitment, surgical specialty, emergency/elective population.
2. Interventions: description of interventions including: components of CGA, healthcare professional delivering intervention, assessment/management tools used (if relevant), time point delivered, duration of time spent with patient, detail of assessment made, detail of care delivered, setting of intervention (eg, clinic, separate ward).
3. Standard care: comparator description, healthcare professionals delivering care in comparator/control group.
4. Outcomes: list of reported outcomes, quantitative data for 11 key areas as defined by Core Outcome Measures in Perioperative and Anaesthetic Care—standard endpoints for perioperative medicine (COMPAC-StEP) working group where possible, including patient comfort, clinical indicators, cognition and stroke, cardiovascular, respiratory, renal, bleeding, morbidity, survival, patient centred outcomes and healthcare resource utilisation.^12^

#### Data sources and search strategy

A search strategy was adapted from a previous Cochrane review.^6^ It includes the themes ‘geriatric care,’ ‘frailty,’ ‘surgery or trauma,’ ‘randomised controlled trials.’ This will be performed across EMBASE, Medline, CINAHL and Cochrane library with help from an information specialist (online supplemental information 2).

10.1136/bmjopen-2021-049875.supp2 Supplementary data

#### Study selection, inclusion and exclusion criteria

Any RCT of CGA versus a control group (standard care) will be included. There will be no age cut-off for the purpose of this review, so that it can identify who has received the intervention, although it is anticipated that studies will include patients 60 years and over.

For the purpose of inclusion, if not otherwise identified as CGA, this study will define perioperative CGA as any review of a patient in the perioperative period by a healthcare professional with training in geriatric medicine (eg, consultant, trainee, specialist nurse). Review exclusively by any other medical professional (eg, anaesthetist or nurse) who is not reported to have received training in geriatric medicine will be excluded.

The perioperative period will be defined as any time between the ‘decision to offer surgery, through to the weeks and months after the procedure’.^13^ Any CGA reported outside of this period will be excluded.

### Study records

#### Data management

Citation management and data collection will be undertaken in Covidence.^14^

#### Selection process

Title and abstracts from all citations identified in the searches will be screened independently for eligibility by two reviewers (RLM, JDB). Screening of full texts will then be undertaken by the same two reviewers. Discrepancies or disagreements in eligibility will be resolved by a third reviewer.

#### Data collection process

Data will be extracted independently by two reviewers using a predefined template developed by the study team. Any discrepancies or disagreements in data extraction will be resolved by a third reviewer.

### Data items

Data items collected include:

1. Participants: sex, age, number randomised, target sample size, reasons for non-recruitment, surgical specialty, emergency/elective population.
2. Interventions: description of interventions including: components of CGA, healthcare professional delivering intervention, assessment/management tools used (if relevant), time point delivered, duration of time spent with patient, detail of assessment made, detail of care delivered, setting of intervention (eg, clinic, separate ward).
3. Standard care: comparator description, healthcare professionals delivering care in comparator/control group.
4. Outcomes: list of reported outcomes, quantitative data for 11 key areas as defined by COMPAC-StEP working group where possible, including patient comfort, clinical indicators, cognition and stroke, cardiovascular, respiratory, renal, bleeding, morbidity, survival, patient centred outcomes and healthcare resource utilisation.^12^

### Risk of bias

Risk of bias at the outcome level for primary outcomes only will be assessed using the Cochrane risk of bias tool, V.2.^15^

### Data synthesis

A narrative synthesis will be presented for all qualitative outcomes. Content analysis will result in detail of the intervention, assessments and outcomes presented in tabulated form, summarising each study side by side as adapted from similar studies.^16 17^ The objectives will be organised according to the definition and domains described in a 1987 conference consensus paper, supplemented with definitions and domains extracted through an iterative process from immersion in the literature.^9^

No meta-analysis will be undertaken as the primary aim of this review is to describe the CGA intervention within each of the trial settings. A simple summary of reported statistics in each trial will be presented.

### Patient and public involvement

There was patient and public involvement in the development of this research question and design of the study via the geriatric perioperative care team at North Bristol National Health Service (NHS) Trust. A formal focus group will be held before publication of the final review.

## Ethics and dissemination

No ethical approval is required for systematic reviews. The study will be disseminated through peer-reviewed manuscript published in a journal and presentation at conferences.
