# Supplementary material for: Comprehensive geriatric assessment in perioperative care: a protocol for a systematic review and qualitative synthesis
Source: BMJ Open. 2021 Dec 28;11(12):e049875. doi: 10.1136/bmjopen-2021-049875 (PMC8719242; doi:10.1136/bmjopen-2021-049875)
Supplement: Supplementary data [file bmjopen-2021-049875supp002.pdf]

CGA medline

1. Geriatric Assessment/

2. geriatric assessment*.tw,kf.

3. Health Services for the Aged/

4. (frail* or sarcopeni* or elder* or senior* or gerontolog* or geriatric* or veteran* or (old* adj (people or person* or resident* or adult* or patient*))).tw,kf.

5. 1 or 2 or 3 or 4

6. ((frail* or sarcopeni* or elder* or senior* or gerontolog* or geriatric* or veteran* or old* people or old* person* or old* resident* or old* adult* or old* patient*) adj3 (assess* or evaluat* or apprais* or function or functioning or comprehensive* or patient care team or patient* education or interprofession* or inter-profession* or interdisciplin* or inter-disciplin* or multi-disciplin* or multidisciplin* or rehab*)).tw,kf.

7. ((frail* or sarcopeni* or elder* or senior* or gerontolog* or geriatric* or veteran* or old* people or old* person* or old* resident* or old* adult* or old* patient*) adj3 (manage* care program* or Critical Pathway* or Program* Evaluation or case manag*)).tw,kf.

8. (geriatric adj3 (evaluation or management or program* or modif* or friendly or intervention or coordinat* or co-ordinat*)).tw,kf.

9. (elder* adj3 (program* or modif* or friendly or intervention* or coordinat* or co-ordinat*)).tw,kf.

10. (acute care for elders or acute care for the elderly or Nurses Improving Care for Healthsystem Elders or modified Hospital Elder Life Program or mHELP or hospitali?ed elder life program*).tw,kf.

11. (geriatrician* or geriatric specialist* or geriatric nurse* or geriatric physician*).tw,kf.

12. (geriatric unit* or geriatric ward*).tw,kf.

13. 6 or 7 or 8 or 9 or 10 or 11 or 12

14. exp Specialties, Surgical/

15. exp surgical procedures, operative/

16. su.fs.

17. Surgery Department, Hospital/

18. perioperative care/ or intraoperative care/ or perioperative nursing/ or postoperative care/ or preoperative care/

19. Trauma Centers/ or General Surgery/

20. (((surgery or surgical) adj (unit* or department* or area*)) or (operating adj (room* or theatre* or theater* or suite*))).mp.

21. (surgery or surgical or trauma or operation or operating or operative).ti,kf.

22. (surgery or surgical or trauma or operation or operating or operative).ab. /freq=2

23. (perioperative or peri operative or intraoperative or intra operative or postoperative or post-operative).ti,ab,kf.

24. hospital*.ti,ab,kf.

25. 14 or 15 or 16 or 17 or 18 or 19 or 20 or 21 or 22 or 23 or 24

26. randomised controlled trial.pt.

27. controlled clinical trial.pt.

28. multicenter study.pt.

29. (randomis* or randomiz* or randomly).ti,ab.

30. groups.ab.

31. (trial or multicenter or multi center or multicentre or multi centre).ti.

32. (intervention? or effect? or impact? or controlled or control group? or (before adj5 after) or (pre adj5 post) or ((pretest or pre test) and (posttest or post test)) or quasiexperiment* or quasi experiment* or pseudo experiment* or pseudoexperiment* or evaluat* or time series or time point? or repeated measur*).ti,ab.

33. 26 or 27 or 28 or 29 or 30 or 31 or 32

34. review.pt.

35. meta analysis.pt.

36. news.pt.

37. comment.pt.

38. editorial.pt.

39. cochrane database of systematic reviews.jn.

40. comment on.cm.

41. (systematic review or literature review).ti.

42. 34 or 35 or 36 or 37 or 38 or 39 or 40 or 41

43. exp Animals/ not Humans/

44. (animal model* or rat or rats or mouse or mice or rodent* or sheep or lambs or murine or pigs or piglets or swine or porcine or rabbit or rabbits or cat or cats or feline or dog or dogs or canine or cattle or bovine or marmoset* or monkey or monkeys or trout or zebra fish*).ti.

45. 42 or 43 or 44

46. 5 and 13 and 25 and 33

47. 46 not 45

CGA embase

1. exp geriatrics/

2. geriatric*.mp.

3. geriatric care/

4. exp geriatrician/

5. esp gerontology/

6. gerontol*.mp.

7. exp frail elderly/

8. aged hospital patient/

9. 1 or 2 or 3 or 4 or 5 or 6 or 7 or 8

10. exp geriatric assessment/

11. "geriatric* assessment*".mp.

12. "comprehensive geriatric* assessment*".mp.

13. "multicomponent assessment*".mp.

14. "multi-component assessment*".mp.

15. "multi-component evaluation".mp.

16. "multi-component evaluation".mp.

17. "multidisciplinary assessment".mp.

18. "multi-disciplinary assessment".mp.

19. "multidisciplinary evaluation".mp.

20. "multi-disciplinary evaluation".mp.

21. (liaison not psychiatry).mp.

22. "hospital* elder life program*".mp.

23. "proactive care of older people".mp.

24. exp geriatric nursing/

25. 10 or 11 or 12 or 13 or 14 or 15 or 16 or 17 or 18 or 19 or 20 or 21 or 22 or 23 or 24

26. exp general surgery/

27. exp surgery/ or exp major surgery/

28. exp geriatric surgery/

29. surg*.mp.

30. laparotomy.mp. or exp laparotomy/

31. preoperative.mp. or exp preoperative evaluation/ or exp preoperative care/ or exp preoperative period/

32. perioperative.mp. or exp perioperative period/

33. esp postoperative care/ or postoperative.mp. or exp postoperative period/

34. 26 or 27 or 28 or 29 or 30 or 31 or 32 or 33

35. 9 and 25 and 34

36. limit 35 to human

37. limit 36 to english language

38. remove duplicates from 37
